# The Effect of Boron Supplementation on Kidney Stones in Patients With Nephrolithiasis: A Double‐Blind Randomized Controlled Trial

**DOI:** 10.1002/fsn3.70777

**Published:** 2025-08-11

**Authors:** Gita Vousoughi, Hamid Soleimanzadeh, Nima Radkhah, Mohammad Asghari Jafarabadi, Fatemeh Bahri, Alireza Ostadrahimi, Vahideh Ebrahimzadeh Attari

**Affiliations:** ^1^ Student Research Committee, Tabriz University of Medical Sciences Tabriz Iran; ^2^ Nutrition Research Center Tabriz University of Medical Sciences Tabriz Iran; ^3^ Cabrini Research Cabrini Health Malvern Victoria Australia; ^4^ School of Public Health and Preventive Medicine Monash University Melbourne Victoria Australia; ^5^ Department of Psychiatry School of Clinical Sciences, Monash University Clayton Victoria Australia; ^6^ Department of Biochemistry and Nutrition School of Nutrition and Food Sciences, Tabriz University of Medical Sciences Tabriz Iran

**Keywords:** boron, kidney stones, nephrolithiasis, randomized controlled trial

## Abstract

Kidney stones (nephrolithiasis) are a common and costly medical condition frequently linked with metabolic disorders. These disorders elevate the risk of serious complications and often necessitate invasive, expensive treatments, highlighting the need for alternative therapies. This study aimed to evaluate the effects of boron supplementation—in the form of boric acid and boron citrate—on kidney stone size and number in patients with nephrolithiasis. In this double‐blind, randomized, placebo‐controlled clinical trial, 60 eligible patients aged 20–65 years with nephrolithiasis were enrolled. Participants were randomly assigned to receive daily oral supplements for 8 weeks: boric acid (10 mg), boron citrate (10 mg), or placebo. The primary outcomes were kidney stone size and number; secondary outcomes included paraclinical parameters. Boron supplementation with either boric acid or boron citrate did not significantly reduce the number of kidney stones in fully adjusted models compared to the control group. The incidence rate ratios (IRR) were 1.09 (95% CI: 0.65–1.85; *p*‐value = 0.740) for boric acid and 1.30 (95% CI: 0.79–2.13; *p*‐value = 0.296) for boron citrate. Quade analysis also revealed no significant differences in kidney stone size among the groups. Regarding laboratory parameters, only urine phosphorus levels were significantly elevated in the boric acid group compared to placebo. No other clinically relevant effects were observed. Despite previous preliminary evidence, this study found no beneficial evidence for boron supplementation in patients with nephrolithiasis.

**Trial Registration:** IRCT20210914052469N1 (www.irct.behdasht.gov.ir)

## Graphical abstract

Boron supplementation showed no benefit for kidney stones in patients with nephrolithiasis (Despite previous preliminary evidence), necessitating further research for definitive conclusions.

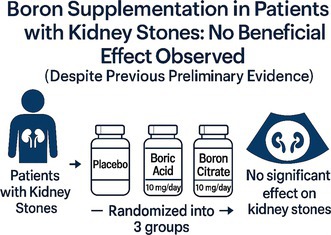

**Abbreviations**
ANCOVA: analysis of covariance
ANOVA: analysis of variance
BMI: body mass index
CONSORT: Consolidated Standards of Reporting Trials
IRCT: Iranian Registry of Clinical Trials
ITT: intention‐to‐treat
RAS: Random Allocation Software
RCT: randomized controlled trial

## 1 Introduction

Kidney stones (Nephrolithiasis) are a common and costly medical issue, affecting nearly 11% of men and 7% of women in the U.S., with annual treatment costs exceeding $2 billion (Shoag et al. 2015; Canvasser et al. 2017). Their formation is often linked to various metabolic disorders, including obesity, diabetes, inflammatory bowel disease, and hypertension (Sigurjonsdottir et al. 2015; Rule et al. 2010; Ye et al. 2023; Taylor et al. 2005; Alexander et al. 2012; Dimke et al. 2021). Additionally, individuals with kidney stones face a heightened risk of adverse health outcomes, like kidney and cardiovascular diseases (Rule et al. 2010; Shastri et al. 2023; Ferraro et al. 2013). These painful stones tend to recur, and patients typically experience unilateral flank pain radiating to the abdomen or inguinal area, a symptom known as renal colic (Shoag et al. 2015; Rule et al. 2020).

Managing nephrolithiasis includes a range of medical interventions, from different pain relievers to surgical options such as shock wave lithotripsy, ureteroscopy, and percutaneous nephrolithotomy (Rule et al. 2020). While effective, these procedures are often invasive and costly, requiring careful postoperative care to ensure optimal recovery and minimize complications (Raheem et al. 2017). Therefore, there is increasing interest in exploring alternative therapies, preventive strategies, lifestyle modifications—particularly nutrition and diet—as well as supplementation or complementary medicine (Jebir and Mustafa 2024; Nirumand et al. 2018).

In this context, preliminary studies suggest that boron supplementation may be a potential intervention. An animal study found that daily boron supplementation significantly reduced the formation of kidney stones (Bahadoran et al. 2016). Additionally, a case study involving a 70‐year‐old woman taking sodium tetraborate before lithotripsy showed improved stone passage and reduced pain and bleeding (Naghii 2013). Another case study of a 46‐year‐old man also supported these findings (Naghii 2014). Furthermore, a pilot study involving 14 patients reported that a weekly boron supplement of 10 mg effectively dissolved or fragmented kidney stones, alleviated pain, and decreased hematuria while also helping to relieve renal colic and ureteral spasms (Naghii et al. 2012).

However, no clinical trials have specifically investigated and confirmed these effects. Therefore, this randomized controlled clinical trial (RCT) aimed to investigate the effects of boron supplementation in the form of boric acid and boron citrate on the number and size of kidney stones in patients with nephrolithiasis.

## 2 Materials and Methods

### 2.1 Study Design

This randomized, double‐blind, placebo‐controlled, parallel‐group clinical trial primarily investigated the effects of boron derivatives (boric acid and boron citrate) on kidney stone size and number in patients with nephrolithiasis. Additionally, the trial evaluated related blood and urine test parameters, as well as associated symptoms and signs.

The present study was registered in the Iranian Registry of Clinical Trials (IRCT) (registration number: IRCT20210914052469N1), and no significant method changes occurred after the trial began. It was conducted under the guidelines outlined in the Declaration of Helsinki, and all procedures involving human subjects received approval from the ethics committee at Tabriz University of Medical Sciences (IR.TBZMED.REC.1400.673). All participants provided written informed consent after being thoroughly informed about the objectives and procedures.

This article was reported following the Consolidated Standards of Reporting Trials (CONSORT) guidelines (Moher et al. 2010).

### 2.2 Study Population and Sample Size

Participants were recruited through advertisements and referrals from urologists in Maragheh, Iran. The study involved individuals aged 20–65 diagnosed with nephrolithiasis by a qualified urologist using ultrasonography. Exclusion criteria included a history of nephrectomy, renal agenesis, kidney failure, cardiovascular and liver diseases, gallstones, cancer, benign prostatic hyperplasia, hypo‐ or hyperthyroidism, urinary tract infections, estrogen therapy, regular use of nutritional supplements or herbal teas, alcohol consumption, pregnancy, breastfeeding, and allergies to boron. Additionally, individuals with indications for immediate surgery or intervention as determined by urologists were excluded.

The sample size was determined based on a previous study (Ardakani Movaghati et al. 2019), which reported kidney stone sizes for the intervention group as 2.66 ± 2.72 mm and for the control group as 5.53 ± 2.91 mm. To achieve at least 80% statistical power with a two‐sided type I error rate of 0.05, the minimum sample size was calculated to be 17 participants per group; however, to account for an anticipated dropout rate of 20%, the sample size was increased to 20 participants per group.

### 2.3 Randomization and Blinding

A research team member not involved in the allocation or outcome assessment generated the allocation sequence using Random Allocation Software (RAS) with a block randomization method at a 1:1:1 ratio. A laboratory technician prepared, packaged, and labeled capsules containing boric acid, boron citrate, or a placebo, identified as A, B, and C. The capsules were made to look, taste, and smell identical. Sequentially numbered opaque sealed envelopes were used to maintain allocation concealment. The allocation sequence was kept confidential from the researcher responsible for enrolling, assessing, and administering the interventions until the trial was finished. Accordingly, participants were randomly assigned to one of three groups: control, boric acid, or boron citrate.

### 2.4 Intervention

Participants were assigned to one of three groups to receive boric acid, boron citrate, or a placebo for 8 weeks. These supplements were prepared at the Nutrition Research Center, Tabriz University of Medical Sciences, Iran. Those in the boric acid group received a boric acid supplement containing 10 mg of elemental boron, while participants in the boron citrate group received a boron citrate supplement with the exact dosage. The control group was given 10 mg of corn starch placebo. Each participant took their assigned capsule once daily, 30 min before lunch or dinner. They were instructed not to change their diet or level of physical activity during the trial and received bi‐weekly phone calls to enhance and monitor adherence. The dosage was determined based on the safe upper intake level and previous studies (Naghii et al. 2012; World Health Organization 1998; Trumbo et al. 2001).

### 2.5 Data Collection

At baseline, demographic and socioeconomic information was collected, along with medical history, smoking history, hormonal issues, alcohol consumption, and details about supplement and drug use. After an overnight fast, height and weight were measured using a Seca stadiometer (Germany) while subjects wore light clothing and were barefoot. Measurements were taken with an accuracy of 0.1 cm and 0.1 kg. Body mass index (BMI) was calculated by dividing weight (kg) by height squared (m^2^).

As the primary outcome, a radiologist assessed the number and size of renal stones through sonographic evaluations conducted before and 8 weeks after the intervention. Additionally, the study examined changes in blood levels of Ca^2+^, phosphorus, urea, creatinine, uric acid, and alkaline phosphatase, as well as urinary levels of calcium, phosphorus, oxalate, citrate, uric acid, and pH. The study investigated changes in blood levels of calcium (Ca^2+^), phosphorus, urea, creatinine, uric acid, and alkaline phosphatase, as well as urinary levels of calcium, phosphorus, oxalate, citrate, uric acid, and pH. To conduct this analysis, 5 mL of fasting venous blood was collected from each participant. The serum was separated and stored at −20°C until further analysis. Additionally, random midstream urine samples were collected in sterile containers. All biochemical analyses were performed using standardized methods at the central laboratory of Maragheh University of Medical Sciences in Maragheh, Iran.

All the outcomes were reported as they were aimed for in the protocol registered in the IRCT.

### 2.6 Compliance and Safety

Participants received phone calls every 2 weeks about their daily capsule intake, any adverse events, and changes to their routines or medications. Moreover, compliance was assessed at the end of the study using cumulative capsule counts, determined by subtracting the number of unused capsules returned from the initial number given. Percentage compliance was then calculated.

### 2.7 Statistical Analysis

Statistical analyses were conducted using IBM SPSS version 26.0 (IBM Corp., Armonk, NY, USA), with a significance threshold set at a two‐tailed *p*‐value of < 0.05. An intention‐to‐treat (ITT) approach was employed to analyze the results, with missing data imputed using the multiple imputation method. In addition, per‐protocol analysis was performed as a sensitivity analysis. The normality of the data was assessed through the Kolmogorov–Smirnov test and Q‐Q plots. Continuous variables were presented as means with standard deviations or medians (25th–75th percentiles), depending on whether they followed a normal or non‐normal distribution, while categorical variables were reported as frequencies and percentages. Kruskal–Wallis and analysis of variance (ANOVA) were applied as appropriate to compare baseline characteristics between the two groups. Analysis of covariance (ANCOVA) was carried out to evaluate changes in blood and urine test results, and the Quade test (a nonparametric ANCOVA) was utilized to assess alterations in kidney stone size. Additionally, Poisson regression was used to analyze changes in the number of kidney stones, and the incidence rate ratio (IRR) was reported accordingly. Baseline values and confounding factors such as age, gender, BMI, and disease duration were adjusted in the mentioned analysis.

## 3 Results

**FIGURE 1:**
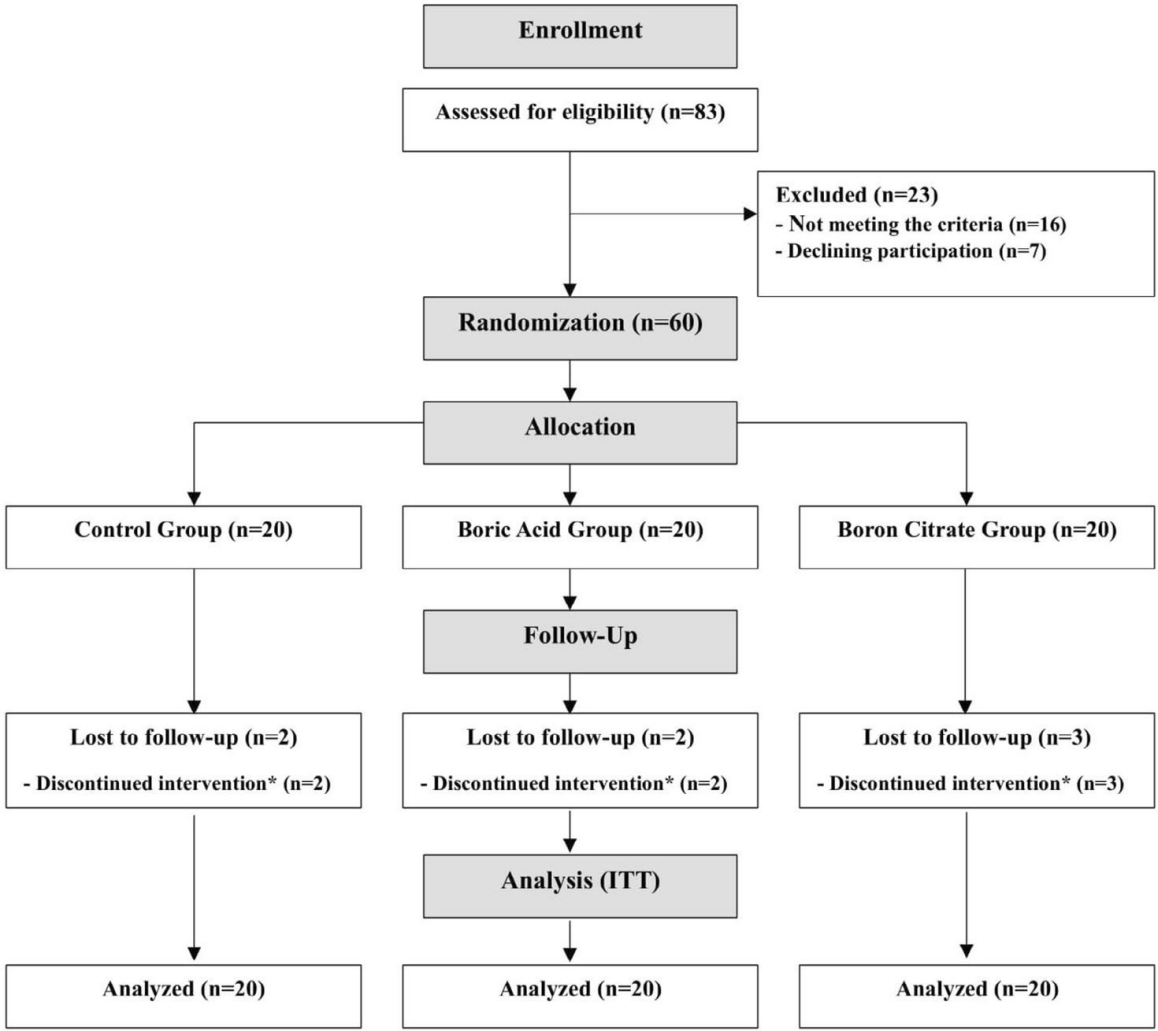
The flow chart of study participants. * Due to the reasons unrelated to the study intervention such as unwillingness, cold weather, or planned trips.

Eighty‐three individuals were initially evaluated based on the inclusion and exclusion criteria between November 2021 and August 2023. Of these, 23 were excluded for not meeting the criteria or for declining participation, resulting in 60 patients with nephrolithiasis for the study. They were randomly assigned to one of three groups: control, boric acid, or boron citrate, with 20 participants in each group undergoing an 8‐week intervention. During this time, two participants from the control group, two from the boric acid group, and three from the boron citrate group discontinued the intervention for reasons unrelated to the study (unwillingness, cold weather, or planned trips). However, all 60 participants were included in the statistical analysis following the ITT approach. The participant flow diagram is shown in Figure 1. The groups had similar demographic factors, including age, gender, weight, BMI, and family history. However, notable differences emerged in terms of education level and disease duration. Participants in the boron groups were more likely to have a higher level of education, while those in the boron citrate group experienced a longer duration of nephrolithiasis (Table 1). Throughout the intervention, patients reported no side effects and achieved a compliance rate of 90%.

**TABLE 1:** Baseline characteristics.

Variables	Control (*n* = 20)	Boric acid (*n* = 20)	Boron citrate (*n* = 20)
Age	45.9 ± 11.68	43 ± 10.87	44.6 ± 11.00
Gender			
Male	14 (70.0%)	14 (70.0%)	13 (65.0%)
Female	6 (30.0%)	6 (30.0%)	7 (35.0%)
Weight (kg)	78.64 ± 18.52	80.40 ± 14.18	76.20 ± 15.17
BMI (kg/m^2^)	29.10 ± 5.46	28.09 ± 3.38	27.52 ± 5.43
Education			
Under diploma	10 (50.0%)	7 (35.0%)	9 (45.0%)
Diploma	9 (45.0%)	8 (40.0%)	7 (35.0%)
University degree	1 (5.0%)	5 (25.0%)	4 (20.0%)
Nephrolithiasis duration			
≤ 5 years	13 (65.0%)	14 (70.0%)	8 (40.0%)
> 5 years	7 (35.0%)	6 (30.0%)	12 (60.0%)
Nephrolithiasis family history			
No	12 (60.0%)	12 (60.0%)	10 (50%)
Yes	8 (40.0%)	8 (40.0%)	10 (50%)

*Note:* Data are presented as Mean ± SD or *n* (%).

### 3.1 Primary Outcomes

Table 2 presents the number of kidney stones before and after the intervention for three groups. At baseline, the groups were similar in number and size of stones. After 8 weeks, the number of kidney stones showed no significant difference in the boric acid and boron citrate groups compared to the control group. Specifically, IRR was as follows: for the boric acid group, IRR (95% CI): 1.13 (0.67–1.90), *p*‐value = 0.645; and for the boron citrate group, IRR (95% CI): 1.25 (0.77–2.03), *p*‐value = 0.370 compared to the control group (Table 2). These results remained consistent even after adjusting for confounding variables, with adjusted IRRs of 1.09 (95% CI: 0.65–1.85), *p*‐value = 0.740 for boric acid and 1.30 (95% CI: 0.79–2.13), *p*‐value = 0.296 for boron citrate (Table 2). Additionally, there were no significant changes in stone size among the groups after the intervention in both models (*p*‐value for *model 1 and model 2* = 977 and 0.916) (Table 3).

**TABLE 2:** Kidney stones number before and after intervention among the three groups.

Variables	Control (*n* = 20)	Boric acid (*n* = 20)	Boron citrate (*n* = 20)
Kidney stone (number)			
Before			
≤ 1	9 (45%)	13 (65%)	11 (55%)
2–3	10 (50%)	6 (30%)	6 (30%)
≥ 4	1 (5%)	1 (5%)	3 (15%)
After			
≤ 1	13 (65%)	14 (70%)	9 (45%)
2–3	7 (35%)	4 (20%)	9 (45%)
≥ 4	0 (0%)	2 (10%)	2 (10%)
Kidney stone (IRR)			
Model 1	1	1.13 (0.67 to 1.90); 0.645*	1.25 (0.77 to 2.03); 0.370*
Model 2	1	1.09 (0.65 to 1.85); 0.740*	1.30 (0.79 to 2.13); 0.296*

*Note:* Data are presented as *n* (%) and IRR (95% CI).

Abbreviation: IRR, incidence rate ratio.

* *p*‐value based on Poisson regression (control group as a reference); adjusted for baseline values (Model 1); adjusted for baseline values, initial BMI, nephrolithiasis duration, age, and gender (Model 2).

**TABLE 3:** Kidney stones size before and after intervention among the three groups.

Variables	Control (*n* = 20)	Boric acid (*n* = 20)	Boron citrate (*n* = 20)	*p*
Stone size (mm)				
Before	5.50 (3.62–6.83)	4.10 (4.00–6.87)	6.17 (3.78–9.25)	0.254^#^
After	5.10 (3.32–6.31)	4.10 (2.62–7.50)	5.50 (3.55–8.75)	0.977*, 0.916**

*Note:* Data are presented as Median (25th–75th centiles).

^#^ *p*‐value for baseline values based on Kruskal–Wallis.

* *p*‐value for change based on Quade test, adjusted for baseline values (Model 1).

** *p*‐value for change based on Quade test, adjusted for baseline values, nephrolithiasis duration, age, and gender (Model 2).

### 3.2 Secondary Outcomes

Table 4 presents the blood levels of calcium (Ca^2+^), phosphorus, urea, creatinine, uric acid, and alkaline phosphatase, as well as the urine levels of calcium, phosphorus, oxalate, citrate, uric acid, and pH levels before and after the intervention for the three groups. Additionally, Table 5 provides a comparison of these parameters among the groups. At baseline, no significant differences were found among the groups, except for alkaline phosphatase levels, which were higher in the control group. After the intervention, urine phosphorus levels significantly increased in the boric acid group compared to the control group, with a mean difference (MD) of 3.70 mEq/L (95% CI: 0.58–6.82) (Table 5). This significance remained after full adjustment, resulting in an MD of 3.40 mEq/L (95% CI: 0.59–6.20). Furthermore, urine phosphorus levels significantly decreased in the boron citrate group compared to the boric acid group, with an MD of −5.09 mEq/L (95% CI: −8.24 to −1.95) (Table 5). This significance also persisted after adjusting for confounding factors, yielding an MD of −4.31 mEq/L (95% CI: −7.13 to −1.48). Additionally, the blood urea level significantly increased in the boron citrate group compared to the boric acid group, with an MD of 3.46 mg/dL (95% CI: 0.08–6.85) (Table 5). This significance remained after adjusting for confounding factors, resulting in an MD of 3.58 mg/dL (95% CI: 0.11–7.05). Other specified blood and urine tests did not significantly change among the three groups, even after controlling for confounding variables (Table 5).

**TABLE 4:** Blood and urine tests before and after intervention among the three groups.

Variables	Control (*n* = 20)	Boric acid (*n* = 20)	Boron citrate (*n* = 20)	*p*
Blood				
Ca^2+^ (mmol/L)				
Before	1.13 ± 0.04	1.12 ± 0.04	1.15 ± 0.09	0.183^#^
After	1.16 ± 0.05	1.12 ± 0.05	1.15 ± 0.06	0.179*, 0.269**
Phosphorus (mg/dL)				
Before	3.67 ± 0.59	3.60 ± 0.47	3.76 ± 0.56	0.666^#^
After	3.49 ± 0.45	3.72 ± 0.56	3.65 ± 0.37	0.188*, 0.171**
Urea (mg/dL)				
Before	32.97 ± 7.70	32.42 ± 6.80	35.07 ± 10.25	0.576^#^
After	31.92 ± 7.82	28.21 ± 4.70	33.66 ± 10.83	0.076*, 0.090**
Creatinine (mg/dL)				
Before	1.07 ± 0.17	1.03 ± 0.10	1.07 ± 0.19	0.682^#^
After	1.08 ± 0.20	1.07 ± 0.16	1.06 ± 0.21	0.586*, 0.431**
Uric acid (mg/dL)				
Before	5.05 ± 1.43	5.13 ± 1.14	4.94 ± 1.15	0.892^#^
After	4.73 ± 1.15	4.45 ± 0.93	4.68 ± 1.11	0.390*, 0.388**
Alkaline phosphatase (U/L)				
Before	246.85 ± 61.23	186.17 ± 47.26	177.55 ± 36.58	< 0.001^#^
After	255.25 ± 51.44	194.71 ± 51.85	212.05 ± 59.50	0.206*, 0.162**
Urine				
Calcium (mg/dL)				
Before	10.82 ± 3.89	12.36 ± 5.66	12.64 ± 4.93	0.451^#^
After	14.18 ± 5.34	12.43 ± 5.33	14.80 ± 5.68	0.343*, 0.347**
Phosphorus (mEq/L)				
Before	32.24 ± 10.30	32.50 ± 7.82	35.52 ± 9.88	0.476^#^
After	27.63 ± 5.52	31.40 ± 6.69	27.08 ± 3.65	0.006*, 0.008**
Oxalate (mmol/L)				
Before	0.22 ± 0.18	0.20 ± 0.12	0.18 ± 0.08	0.553^#^
After	0.15 ± 0.06	0.17 ± 0.07	0.18 ± 0.07	0.514*, 0.696**
Citrate (mmol/L)				
Before	1.86 ± 1.22	2.17 ± 0.90	2.28 ± 1.24	0.485^#^
After	1.65 ± 0.75	1.49 ± 0.56	1.62 ± 0.41	0.632*, 0.711**
Uric acid (mg/dL)				
Before	48.47 ± 10.15	41.94 ± 12.83	44.88 ± 9.53	0.176^#^
After	44.63 ± 12.17	42.68 ± 9.93	44.03 ± 7.09	0.983*, 0.973**
pH				
Before	5.30 ± 0.55	5.30 ± 0.50	5.27 ± 0.55	0.985^#^
After	5.05 ± 0.22	5.15 ± 0.49	5.00 ± 0.02	0.298*, 0.255**

*Note:* Data are presented as Mean ± SD.

^*#*^ *p*‐value for baseline based on analysis of variance (ANOVA).

* *p*‐value for change based on ANCOVA, adjusted for baseline values (Model 1).

** *p*‐value for change based on ANCOVA, adjusted for baseline values, initial BMI, age, and gender (Model 2).

**TABLE 5:** Comparison between three groups for changes in blood and urine tests.

Variables	Boric acid vs. Control	Boron citrate vs. Control	Boron citrate vs. Boric acid
Blood			
Ca^2+^ (mmol/L)			
Model 1	−0.029 (−0.059 to 0.002)	−0.016 (−0.047 to 0.015)	−0.013 (−0.018 to 0.044)
Model 2	−0.025 (−0.056 to 0.006)	−0.012 (−0.043 to 0.020)	−0.014 (−0.018 to 0.045)
Phosphorus (mg/dL)			
Model 1	0.26 (−0.02 to 0.54)	0.14 (−0.14 to 0.42)	−0.12 (−0.40 to 0.16)
Model 2	0.27 (−0.01 to 0.56)	0.16 (−0.13 to 0.45)	−0.11 (−0.40 to 0.17)
Urea (mg/dL)			
Model 1	−3.30 (−6.65 to 0.06)	0.17 (−3.21 to 3.54)	**3.46 (0.08 to 6.85)**
Model 2	−3.06 (−6.53 to 0.41)	0.52 (−3.01 to 4.05)	**3.58 (0.11 to 7.05)**
Creatinine (mg/dL)			
Model 1	0.02 (−0.07 to 0.11)	−0.02 (−0.11 to 0.06)	−0.05 (−0.14 to 0.04)
Model 2	0.05 (−0.26 to 0.12)	0.02 (−0.05 to 0.09)	−0.03 (−0.10 to 0.04)
Uric acid (mg/dL)			
Model 1	−0.31 (−0.85 to 0.22)	0.01 (−0.52 to 0.55)	0.33 (−0.21 to 0.87)
Model 2	−0.13 (−0.60 to 0.33)	0.18 (−0.28 to 0.64)	0.32 (−0.14 to 0.77)
Alkaline phosphatase (U/L)			
Model 1	−19.45 (−50.27 to 11.37)	3.73 (−28.05 to 35.52)	23.19 (−4.29 to 50.66)
Model 2	−15.89 (−44.57 to 12.78)	8.31 (−21.21 to 37.84)	24.21 (−1.25 to 49.67)
Urine			
Calcium (mg/dL)			
Model 1	−2.05 (−5.51 to 1.42)	0.27 (−3.20 to 3.75)	2.32 (−1.12 to 5.75)
Model 2	−2.18 (−5.75 to 1.39)	0.11 (−3.48 to 3.70)	2.29 (−1.22 to 5.81)
Phosphorus (mEq/L)			
Model 1	**3.70 (0.58 to 6.82)**	−1.39 (−4.54 to 1.76)	**−5.09 (−8.24 to −1.95)**
Model 2	**3.40 (0.59 to 6.20)**	−0.91 (−3.76 to 1.94)	**−4.31 (−7.13 to −1.48)**
Oxalate (mmol/L)			
Model 1	0.02 (−0.02 to 0.06)	0.02 (−0.02 to 0.07)	0.00 (−0.04 to 0.05)
Model 2	0.02 (−0.03 to 0.06)	0.02 (−0.03 to 0.06)	0.00 (−0.04 to 0.04)
Citrate (mmol/L)			
Model 1	−0.17 (−0.55 to 0.20)	−0.04 (−0.43 to 0.34)	0.13 (−0.25 to 0.51)
Model 2	−0.15 (−0.53 to 0.22)	−0.05 (−0.43 to 0.33)	0.10 (−0.27 to 0.47)
Uric acid (mg/dL)			
Model 1	0.03 (−6.15 to 6.215)	0.49 (−5.56 to 6.54)	0.46 (−5.58 to 6.49)
Model 2	0.07 (−6.37 to 6.506)	0.66 (−5.65 to 7.0)	0.59 (−5.63 to 6.81)
pH			
Model 1	0.10 (−0.10 to 0.30)	−0.05 (−0.25 to 0.14)	−0.15 (−0.35 to 0.04)
Model 2	0.11 (−0.09 to 0.31)	−0.05 (−0.25 to 0.15)	−0.16 (−0.36 to 0.04)

*Note:* Data are reported as MD (95% CI). Model 1: adjusted for baseline values; Model 2: adjusted for baseline values, initial BMI, age, and gender. Bold values are statistically significant (*p*‐value < 0.05).

## 4 Discussion

The current RCT found no significant effect of boron supplementation (boric acid and boron citrate) on kidney stone size or number compared to the control group. As for secondary outcomes, there were no significant changes in blood levels of calcium, phosphorus, creatinine, uric acid, or alkaline phosphatase, nor in urine levels of calcium, oxalate, citrate, uric acid, or pH. However, urine phosphorus levels significantly increased in the boric acid group compared to the control group. Notably, urine phosphorus levels were significantly decreased in participants receiving boron citrate compared to those receiving boric acid, and blood urea levels increased in those taking boron citrate compared to those taking boric acid. As a sensitivity analysis, a per‐protocol analysis was also performed (data not shown), and the results were consistent with the ITT analysis. Our findings, however, appear inconsistent with prior evidence.

An animal study conducted by Bahadoran et al. reported that boron supplementation, whether combined with vitamin E or administered separately, may offer a protective effect against the formation of kidney stones (Bahadoran et al. 2016). Additionally, two case reports indicated that boron supplementation facilitated the complete removal of stones or their disposal without hydronephrosis, along with significant pain relief and a marked reduction in ureteral bleeding or hematuria (Naghii 2013, 2014). A pilot study involving 14 patients also demonstrated that boron supplementation (10 mg/week) effectively dissolved or fragmented kidney stones, alleviated pain, decreased hematuria, and helped relieve renal colic and ureteral spasms (Naghii et al. 2012).

Moreover, studies have indicated that boron may influence kidney stone formation by modulating calcium and magnesium metabolism and affecting the urinary excretion of other nephrolithiasis‐related minerals. Meacham et al. discovered that 10 months of boron supplementation (3 mg/day) reduced urinary calcium and magnesium excretion among athletes while also increasing phosphorus excretion (Meacham et al. 1995, 1994). Hunt et al. further reported that boron supplementation reduced urinary oxalate excretion in postmenopausal women with low magnesium levels, underscoring its potential role in preventing kidney stones (Nielsen et al. 1987; Hunt et al. 1997).

The mechanism behind these effects has been investigated in different studies, suggesting that boron, due to its antioxidant properties, may have a protective effect against kidney stone formation (Bahadoran et al. 2016; Naghii, Eskandari, et al. 2014). In addition, studies point out that boron may affect kidney stone formation due to its significant role in steroid hormones, such as vitamin D and sex hormones (Naghii et al. 2011; Miljkovic et al. 2004; Hu et al. 2017). Testosterone may negatively affect stone formation by decreasing renal osteopontin expression and increasing urinary oxalate excretion. In contrast, estrogen prevents stone formation by enhancing osteopontin expression and decreasing urinary oxalate levels (Naghii, Babaei, and Hedayati 2014; Gupta et al. 2016; Rahman et al. 2022). Studies indicate that inhibiting microsomal enzymes responsible for hydroxylating steroids may allow boron to elevate levels of 17β‐estradiol, possibly via interactions with serine protease enzymes. This mechanism could foster a more favorable balance between testosterone and estrogen, which might influence the formation and prevention of kidney stones (Naghii et al. 2011; Miljkovic et al. 2004).

This study did not confirm the findings of the mentioned articles. However, it is important to note that this is the first randomized controlled trial of its kind; the absence of comparable clinical trials limits direct comparisons with our results. In addition, most cited studies are based on animal research, case reports, or pilot studies, which may lead to overestimation and could not provide conclusive evidence. Moreover, the optimal dosage for boron supplementation is still a topic of debate. In our study, we used 10 mg/day of elemental boron, which is below levels that could cause acute toxicity but may not be sufficient for achieving a clinical effect. Additionally, the bioavailability and pharmacokinetics of boron might not correspond to the doses used in our trial (Institute of Medicine (US) Panel on Micronutrients 2001).

Another crucial factor is the individual variability in responses to supplementation. Kidney stones are complex conditions influenced by various factors, such as genetic predispositions, diet, hydration status, urinary pH, and underlying conditions like hypercalciuria or hyperoxaluria (Howles and Thakker 2020; D'Ambrosio et al. 2022). Consequently, these factors may have masked any potential benefits, highlighting the need for additional clinical studies on boron supplementation in diverse populations, considering different stone types to enable comparisons and draw more definitive conclusions.

This study, like any research, has its strengths and limitations. We employed two forms of boron supplementation within the RCT to evaluate and compare their effects. Although the sample size was carefully calculated, larger studies could offer more robust evidence by better controlling for confounding factors and examining effect modifiers. Despite some dropouts, each group retained adequate sample sizes, and we employed an ITT approach to ensure unbiased comparisons between treatment groups and reduce the impact of dropouts. We thoroughly investigated nephrolithiasis‐related minerals and parameters in blood and urine. However, our analysis relied on random urine samples, and a 24‐h collection could yield a more accurate assessment. Additionally, since this is the first study investigating the effects of boron on kidney stones, we did not stratify by the type of kidney stone, which should be considered in future studies.

## 5 Conclusion

The study found that boron supplementation with boric acid and boron citrate had no significant effect on kidney stone size or number, blood levels of calcium, phosphorus, urea, creatinine, uric acid, or alkaline phosphatase, or urine levels of calcium, oxalate, citrate, uric acid, or pH compared to the control group. Notably, urine phosphorus levels increased in the boric acid group compared to the control group, which should be evaluated in those with calcium phosphate stones. Although this study found no benefits of boron supplementation regarding kidney stones and related paraclinical parameters, it is important to note that it represents the first randomized controlled trial on this topic. Therefore, additional research is warranted to establish conclusive evidence.

## Author Contributions

**Gita Vousoughi:** conceptualization (equal), investigation (equal), methodology (equal), writing – original draft (equal), writing – review and editing (equal). **Hamid Soleimanzadeh:** methodology (equal). **Nima Radkhah:** methodology (equal), writing – original draft (equal), writing – review and editing (equal). **Mohammad Asghari Jafarabadi:** methodology (equal), software (equal). **Fatemeh Bahri:** writing – original draft (equal). **Alireza Ostadrahimi:** conceptualization (equal), investigation (equal), methodology (equal), project administration (equal), writing – original draft (equal), writing – review and editing (equal). **Vahideh Ebrahimzadeh Attari:** conceptualization (equal), investigation (equal), methodology (equal), project administration (equal), writing – original draft (equal), writing – review and editing (equal).

## Ethics Statement

The present study was registered in the Iranian Registry of Clinical Trials (IRCT) (registration number: IRCT20210914052469N1). It was conducted under the guidelines outlined in the Declaration of Helsinki, and all procedures involving human subjects received approval from the ethics committee at Tabriz University of Medical Sciences (IR.TBZMED.REC.1400.673). All participants provided written informed consent after being thoroughly informed about the objectives and procedures.

## Conflicts of Interest

The authors declare no conflicts of interest.

## Data Availability

Data described in the manuscript will be made available upon request.
